# Status and determinants of health services utilization among elderly migrants in China

**DOI:** 10.1186/s41256-018-0064-0

**Published:** 2018-03-21

**Authors:** Xiaofang Zhang, Bin Yu, Tiantian He, Peigang Wang

**Affiliations:** 1 School of Health Sciences, Wuhan University, Wuhan, China; 2 Department of Epidemiology, University of Florida, Gainesville, Florida USA

**Keywords:** Elderly migrants, Health services utilization, Influential factors

## Abstract

**Background:**

The household registration system in China places migrants in a vulnerable status regarding access to local public services, including limited access to health services. Most studies on migrants’ health services utilization targeted on working-age migrants, and there has been a paucity of studies conducted among elderly migrants. This study aims to investigate the status of health services utilization and its influential factors among elderly migrants.

**Methods:**

Data (13,043 participants, 52.4% male, mean age 66.22 ± 6.20) were derived from the 2015 Migrant Dynamics Monitoring Survey. The outcome variable in the study was health services utilization, consisting of doctor visits, hospitalization and local inpatient care. The Behavioral Model of Health Service Use was applied to categorize the influential factors into three components, including predisposing, enabling and need factors. Multivariate logistic regression analysis was used to investigate the influential factors of the three components of health services utilization.

**Results:**

Of the total sample, 45.5% would visit a doctor when they were ill, 81.8% would prefer to be hospitalized when recommended by doctors, and 71.6% (those who were hospitalized) would choose to receive local inpatient care rather than going back to their hometown. Age, marital status, household income, years of residence, migration range, reasons for migration, size of friend network, health insurance type, local health insurance status and chronic disease status were significantly associated with health services utilization.

**Conclusion:**

A low level of local health services utilization was observed among elderly migrants. Enabling factors played important roles in promoting health services utilization among elderly migrants. Policy and decision makers may consider improving the capability for elderly migrants to access health services, such as increasing income and providing local health insurance.

## Background

### Migration in the world and in China

With the advancement of industrialization and urbanization, a growing number of people are migrating from rural areas to urban areas or from less developed countries to more developed countries [1, 2]. According to a World Bank report, more than 247 million people migrated from their home country to another country, with 750 million migrating within countries [3]. The large number of migrants presents a global and public health challenge for health policy makers and practitioners. In the past two decades, more Chinese people have migrated from rural areas to urban areas to pursue a higher quality of life. According to the sixth nationwide population census in 2010, the number of rural-to-urban migrants increased to 260 million, accounting for 19.5% of the total population. Meanwhile, the number of elderly migrants aged 60 years and older reached 9.34 million [4]. With the aging of the national population and the rising trend of entire family migration, the number of elderly migrants is anticipated to increase continuously in the future.

### Challenging life of elderly migrants in urban areas

The quality of life among elderly migrants in the urban areas is challenging, and requires more attention from researchers and policy makers. In general, most elderly migrants do not have a steady income to support themselves as well as their family. Economically, they are partially or fully dependent on their children. However, a considerable number of the elderly are still active in the labor market with extremely disadvantaged positions [5]. Most of them are occupied in labor-intensive work with long working hours, poor working environment, and low payment, such as manufacturing or catering fields [6]. Elderly migrants also have experienced much difficulty in integrating themselves into urban life [2, 7, 8]. There are several possible reasons. First, migrants leave their familiar social environment in the place of origin with a loss of social capital and face a series of problems due to differences in language, living habits, poor ability to accept new things, social capital reconstruction, and obstacles to interpersonal communication [9, 10]. Second, most elderly migrants live with their children and may experience more conflicts with inter-generation interactions. Especially in families with low socio-economic status, there is always an imbalance between taking care of the elderly and supporting the young, and in most situations, the needs of the elderly are usually ignored. These challenges and difficulties exert negative effects on the health of the elderly migrants [5, 11].

### Limited health care services among elderly migrants

As the physiological functions of elderly migrants deteriorate with age, they are at high risk of health-related problems, such as cancer, fractures, hypertension and diabetes [12], leading to a great need of health care services in the urban area. Nevertheless, their migrant status in the household registration system has prevented them from enjoying the same social welfare as urban residents. In addition, without local health insurance, elderly migrants have limited access to health care services considering the high costs and inconvenience in obtaining insurance reimbursement [13, 14]. Surveys conducted among migrant workers indicated that the utilization of health services was at a low level both in the areas where migrants originated from and in their new locations [15]. Another study conducted in Shenzhen, China, indicated that 55.1% of migrant workers did not have health insurance and that 62.1% did not visit a doctor when they were ill [16]. As the number of elderly migrants continues to grow, the utilization of health care services among elderly migrants in urban areas is of great significance for public health and social harmony. However, most studies on health care services utilization among migrants have mostly focused on workers ranging from 20 to 50 years old, and few studies have paid attention to the elderly migrants. One purpose of the study is to investigate the status of health care services utilization among elderly migrants by using national survey data.

### Behavioral model of health service use

The Behavioral Model of Health Service Use, which was put forward by Anderson at the University of Chicago in 1968, has received international recognition in explaining influential factors of health services utilization [17]. This model explores factors from three components: predisposing factors, enabling factors and need factors. Predisposing factors refer to individual sociodemographic characteristics that influence recognition of health problems and need for health services, such as gender, age and education. Enabling factors include individual and community resources that facilitate the use of services, such as income, insurance coverage, social support from family and friends, and accessibility of services. Need factors include the assessment of one’s health condition, objective and professional evaluation of need (e.g., diagnosed diseases), and a subjective assessment (e.g., self-rated health). These three components are integrated together, influencing the utilization of health care services. Many scholars around the world and in China have applied this model to investigate health services utilization among different populations.

A study based on the 2013 Migrant Dynamics Monitoring Survey Data in China indicated that predisposing factors (e.g., gender, marital status), and enabling factors (e.g., the duration of stay in the city of residence and local health insurance) exerted significant effect on health services utilization. Married females who have lived in the area for a long time and have local health insurance were more likely to visit a doctor when they were ill [18]. Another study using the Korea Health Panel Survey data indicated that need factors, such as chronic diseases, were important determinants of using physician and inpatient hospital services among Koreans [19]. Further, one study conducted among first generation Afghan migrants in Istanbul found that the determinants of health care services utilization were income and other enabling factors, such as family presence in Turkey [20]. However, to our knowledge, no study has applied the Behavioral Model to investigate health care utilization among elderly migrants in China.

### Purpose of study

This study aims to employ the Behavioral Model of Health Service Use to investigate the status and determinants of health services utilization among elderly migrants. The ultimate purpose is to provide evidence for developing future effective interventions and potential policies to improve access to health services for vulnerable populations.

## Methods

### Data source and study sample

Data in the study were derived from the 2015 Migrant Dynamics Monitoring Survey, an annual nationwide cross-sectional survey sponsored by the National Health and Family Planning Commission. The purpose of the survey was to examine the socioeconomic status of migrants, public health services utilization and family planning management and services. The participants in this survey were migrants aged 15 years and older, who did not have local *Hukou* and had been residing at their current location for more than one month. The participants were selected by adopting a three-stage stratified probability proportionate to size (PPS) sampling strategy. Overall, 348 cities and 10,300 communities were selected from 31 provinces and Xinjiang production and construction corps based on the PPS method. Twenty eligible individual migrants were randomly selected in each selected community. A total of 206,000 participants were recruited. Signed informed consent was collected. For the purpose of this study, only migrants aged 60 years and older (born before May, 1955) were included, yielding a final sample of 13,043, with a return rate of 100.0%.

The survey was conducted using a paper-and-pencil, self-administered questionnaire, and the participants returned the questionnaire to the data collector after completion. The survey was anonymous and confidential, and the participants had the right to refuse to answer questions or withdraw from the survey.

### Variables

#### Outcome variable

Three outcome variables were used to measure health services utilization, including doctor visits, hospitalization and local inpatient care. Doctor visits were measured using the question, “How do you deal with diseases with minor symptoms in daily life?” The answer options included “1 = See a doctor”, and “0=Not to see a doctor (self-medication or not taking any measures)”. In the question, “minor symptoms” referred to symptoms that make the patient feel uncomfortable but do not have a serious effect on daily life. Hospitalization was measured using the question, “In the past year, did you get inpatient care when you were recommended to be hospitalized by doctors?” with an answer option of “1 = Yes”, and “0 = No”. Local inpatient care was measured using the question, “If you were hospitalized, where did you get the treatment?” with answer options of “1 = Local hospital”, and “0 = Hospital in hometown”.

### Predisposing factors

Predisposing factors included age (in years), gender (male/female), marital status (currently married/single, single including unmarried, divorced and widowed), *Hukou* (rural residence/urban residence) and education (elementary school and below/middle school/high school and above).

#### Enabling factors

Enabling factors included monthly household income (less than 3500 yuan/3501–7000 yuan/more than 7000 yuan), migration characteristics, and health insurance status. Migration characteristics were measured by duration of migration (in years), the range of migration (across provinces/across cities within a province/across counties within a city), and reasons for migration (seeking jobs/looking after children or grandchildren/ retirement or keeping fit/others). Health insurance status included health insurance types (New Rural Cooperative Medical Scheme (NCMS), Urban Employment Medical Insurance (UEMI), Urban Residents Medical Insurance (URMI), Cooperative Medical Insurance for Urban and Rural Residents (CMIURR) and free health services), and locations where they were enrolled in insurance. According to the difference in locations where they were enrolled in insurance, health insurance was categorized into local health insurance and hometown health insurance.

#### Need factors

Need factors included chronic disease status and self-rated health status. Chronic disease status was measured using the question, “Did you suffer from hypertension or diabetes diagnosed by physicians?” with options of “0 = No”, and “1 = Yes”. Self-rated health status was measured by asking “How do you feel about your health?” with answer options of “1 = Poor”, “2 = Neutral” and “3 = Good”.

### Statistical analysis

Descriptive statistics (frequency, percentage, mean (SD)) were used to describe the characteristics of the study sample. A Chi-square test was used to analyze the difference of health services utilization among elderly migrants with different characteristics. Multivariate logistic regression analysis was employed to further explore factors influencing health services utilization. Statistical analyses were conducted using SPSS, version 21.0 (IBM, Armonk, North Castle, NY).

## Results

### Characteristics of the study sample

The mean age of the sample was 66.22 (SD = 6.20) years old and 52.4% were males. Most participants were married (81.5%), had an education attainment of middle school level and below (85.4%), and indicated legal rural residences (67.0%). The duration of migration was 6.15 (SD = 6.51) years on average. The proportion of across-province migration was the highest (42.8%), followed by across cities within a province migration (31.8%), and across counties within a city migration (25.4%). Three main reasons for movement were taking care of children or grandchildren (34.0%), retirement/keeping fit (32.8%) and seeking jobs (23.4%). Overall, 92.2% of the participants had one type of health insurance. Additionally, 22.2% of the participants suffered from hypertension or diabetes. Self-reported health status of poor, neutral and good were 10.9%, 43.9%, 45.2%, respectively (Table 1).

**Table 1:** Characteristics of the elderly migrants in China, n(%)

Variables	Male	Female	Total
Total, n (%)	6838 (52.4%)	6205 (47.6%)	13,043 (100%)
Age (years)			
Mean (SD)	66.18 (6.09)	66.26 (6.31)	66.22 (6.20)
Marital status			
Single	873 (12.8%)	1546 (24.9%)	2419 (18.5%)
Currently married	5965 (87.2%)	4659 (75.1%)	10,624 (81.5%)
*Hukou* status			
Rural residence	4550 (66.5%)	4195 (67.6%)	8745 (67.0%)
Urban residence	2288 (33.5%)	2010 (32.4%)	4298 (33.0%)
Education			
Elementary school and below	3527 (51.6%)	4272 (68.8%)	7799 (59.8%)
Middle school	2097 (30.7%)	1239 (20.0%)	3336 (25.6%)
High school and above	1214 (17.8%)	694 (11.2%)	1908 (14.6%)
Monthly family Income (RMB)			
Mean (SD)	6303.34 (7689.11)	6724.54 (14,763.11)	6503.72 (11,606.68)
Years of living in the city of residence			
Mean (SD)	6.33 (6.68)	5.95 (6.32)	6.15 (6.51)
Migration range			
Across provinces	2913 (42.6%)	2674 (43.1%)	5587 (42.8%)
Across cities within a province	2187 (32.0%)	1957 (31.5%)	4144 (31.8%)
Across counties within a city	1736 (25.4%)	1574 (25.4%)	3310 (25.4%)
Flow reasons			
Seeking jobs	2226 (32.6%)	826 (13.3%)	3052 (23.4%)
Looking after children or grandchildren	1956 (28.6%)	2483 (40.0%)	4439 (34.0%)
Retirement/Keeping fit	2037 (29.8%)	2246 (36.2%)	4283 (32.8%)
Others	619 (9.1%)	650 (10.5%)	1269 (9.7%)
Health insurance			
Uninsured	516 (7.5%)	502 (8.1%)	1018 (7.8%)
Underinsured	6322 (92.5%)	5703 (91.9%)	12,025 (92.2%)
Chronic diseases			
Yes	1368 (20.0%)	1530 (24.7%)	2898 (22.2%)
No	5470 (80.0%)	4675 (75.3%)	10,145 (77.8%)
Self-rated health			
Poor	654 (9.6%)	770 (12.4%)	1424 (10.9%)
Neutral	2908 (42.5%)	2821 (45.5%)	5729 (43.9%)
Good	3276 (47.9%)	2614 (42.1%)	5890 (45.2%)

### Health services utilization

The results in Table 2 indicate that when the elderly migrants felt ill, 45.5% would see a doctor, 52.5% would buy medicine in local dispensaries or bring medicine from hometown and 2.0% would not take any measures.

**Table 2:** Health services utilization among elderly migrants in China, n (%)

Variables	Male	Female	Total
Health-seeking Behavior for Diseases with Minor Symptoms			
Doctor visits	3030 (44.3%)	2904 (46.8%)	5934 (45.5%)
Self-medication	3664 (53.6%)	3183 (51.3%)	6847 (52.5%)
Not taking any measures	144 (2.1%)	118 (1.9%)	262 (2.0%)
Hospitalization if Recommended			
Yes	533 (81.5%)	516 (82.0%)	1049 (81.8%)
No	121 (18.5%)	113 (18.0%)	234 (18.2%)
Inpatient Care			
Local hospital	372 (69.8%)	379 (73.4%)	751 (71.6%)
Hometown hospital	96 (18.0%)	93 (18.0%)	189 (18.0%)
Others	65 (12.2%)	44 (8.5%)	109 (10.4%)

In the past 12 months, 1283 (9.8%) of the elderly migrants were recommended to be hospitalized by their doctors. Among them, 81.8% received inpatient care and 18.2% did not. As for the reasons why they were not hospitalized, responses of hospitalization being unnecessary, economic hardship and inconvenience of obtaining insurance reimbursement accounted for 40.2%, 22.2% and 12.0%, respectively. Among the elderly who received inpatient care, 71.6% chose local hospitals, 18.0% chose hometown hospitals and 10.4% had been hospitalized in both places (current city and hometown) or other places.

### Factors associated with health services utilization

Chi-square test results in Table 3 indicate that the older, female, single migrants with urban residence, high school education or above, higher monthly household income, short duration of migration, migration across provinces, migration to take care of children or grandchildren or illness treatment/retirement, more friends in current residence, local health insurance, and with chronic diseases were more likely to visit a doctor with minor symptoms. People with higher household income, migration for looking after children or grandchildren and with chronic diseases were more likely to be hospitalized when recommended by doctors. People with migration time longer than 10 years, migration across counties within a city, more friends in current residence, and with local health insurance had a higher probability of receiving local inpatient care.

**Table 3:** Univariate analysis of health services utilization among elderly migrants in China, n(%)

Variables	Doctor visits		Hospitalization		inpatient care	
	Yes	No	Yes	No	Local hospitals	Hometown hospitals
Predisposing factors						
Age (year)						
60–64	2916 (43.4%)	3800 (56.6%) *	383 (81.7%)	86 (18.3%)	263 (77.1%)	78 (22.9%)
65–69	1458 (46.2%)	1699 (53.8%)	263 (81.7%)	59 (18.3%)	184 (78.6%)	50 (21.4%)
≥ 70	1560 (49.2%)	1610 (50.8%)	403 (81.9%)	89 (18.1%)	304 (83.3%)	61 (16.7%)
Gender						
Male	3030 (44.3%)	3808 (55.7%) *	533 (81.5%)	121 (18.5%)	372 (79.5%)	96 (20.5%)
Female	2904 (46.8%)	3301 (53.2%)	516 (82.0%)	113 (18.0%)	379 (80.3%)	93 (19.7%)
Marital status						
Single	1200 (49.6%)	1219 (50.4%)*	243 (81.5%)	55 (18.5%)	180 (81.1%)	42 (18.9%)
Currently married	4734 (44.6%)	5890 (55.4%)	806 (81.8%)	179 (18.2%)	571 (79.5%)	147 (20.5%)
Hukou status						
Rural residence	3911 (44.7%)	4834 (55.3%)*	678 (81.2%)	157 (18.8%)	476 (78.2%)	133 (21.8%)
Urban residence	2023 (47.1%)	2275 (52.9%)	371 (82.8%)	77 (17.2%)	275 (83.1%)	56 (16.9%)
Education						
Elementary/less	3545 (45.5%)	4254 (54.5%)*	675 (82.1%)	147 (17.9%)	479 (78.5%)	131 (21.5%)
Middle school	1468 (44.0%)	1868 (56.0%)	222 (80.1%)	55 (19.9%)	159 (82.8%)	33 (17.2%)
High school/above	921 (48.3%)	987 (51.7%)	152 (82.6%)	32 (17.4%)	113 (81.9%)	25 (18.1%)
Enabling factors						
Monthly household income (RMB)						
≤ 3500 yuan	1663 (39.7%)	2524 (60.3%)*	335 (80.9%)	79 (19.1%)*	250 (84.2%)	47 (15.8%)*
3501–7000 yuan	2518 (45.8%)	2979 (54.2%)	463 (79.3%)	121 (20.7%)	330 (79.1%)	87 (20.9%)
> 7000 yuan	1753 (52.2%)	1606 (47.8%)	251 (88.1%)	34 (11.9%)	171 (75.7%)	55 (24.3%)
Years of living in the city of residence						
≤ 4 years	3146 (46.3%)	3646 (53.7%)*	513 (80.7%)	123 (19.3%)	346 (75.1%)	115 (24.9%)*
5–9 years	1496 (46.0%)	1755 (54.0%)	257 (82.4%)	55 (17.6%)	182 (78.1%)	51 (21.9%)
≥ 10 years	1292 (43.1%)	1708 (56.9%)	279 (83.3%)	56 (16.7%)	223 (90.7%)	23 (9.3%)
Migration range						
Across provinces	2703 (48.4%)	2884 (51.6%)*	380 (81.2%)	88 (18.8%)	268 (76.4%)	83 (23.6%)*
Across cities within a province	1835 (44.3%)	2309 (55.7%)	321 (81.5%)	73 (18.5%)	227 (79.6%)	58 (20.4%)
Across counties within a city	1396 (42.2%)	1914 (57.8%)	348 (82.7%)	73 (17.3%)	256 (84.2%)	48 (15.8%)
Flow reasons						
Seeking jobs	1184 (38.8%)	1868 (61.2%)*	129 (73.7%)	46 (26.3%)*	87 (86.1%)	14 (13.9%)*
Looking after children or grandchildren	2114 (47.6%)	2325 (52.4%)	319 (85.8%)	53 (14.2%)	215 (73.1%)	79 (26.9%)
Retirement/keeping fit	2022 (47.2%)	2261 (52.8%)	519 (82.3%)	112 (17.7%)	396 (83.0%)	81 (17.0%)
Others	614 (48.4%)	655 (51.6%)	82 (78.1%)	23 (21.9%)	53 (77.9%)	15 (22.1%)
Friends number						
< 5	2390 (43.3%)	3130 (56.7%)*	493 (81.2%)	114 (18.8%)	335 (74.6%)	114 (25.4%)*
≥ 5	3544 (47.1%)	3979 (52.9%)	556 (82.2%)	120 (17.8%)	416 (84.7%)	75 (15.3%)
Health insurance						
NRCMS	3003 (44.8%)	3693 (55.2%)	543 (81.4%)	124 (18.6%)	373 (76.0%)	118 (24.0)*
UEMI	1095 (45.2%)	1325 (54.8%)	233 (85.0%)	41 (15.0%)	171 (81.4%)	39 (18.6%)
Others (URMI, CMIURR and free health service)	946 (47.3%)	1053 (52.7%)	165 (83.3%)	33 (16.7%)	132 (88.6%)	17 (11.4%)
Location enrolled health insurance						
Hometown	4339 (44.5%)	5416 (55.5%)*	811 (82.4%)	173 (17.6%)	566 (77.2%)	167 (22.8%)*
Local	665 (52.5%)	601 (47.5%)	121 (84.0%)	23 (16.0%)	105 (94.6%)	6 (5.4%)
Need factors						
Chronic diseases						
Yes	1403 (48.4%)	1495 (51.6%)*	553 (84.0%)	105 (16.0%)*	415 (84.3%)	77 (15.7%)*
No	4531 (44.7%)	5614 (55.3%)	496 (79.4%)	129 (20.6%)	336 (75.0%)	112 (25.0%)
Self-rated health						
Poor	645 (45.3%)	779 (54.7%)	404 (81.8%)	90 (18.2%)	295 (84.3%)	55 (15.7%)*
Neutral	2545 (44.4%)	3184 (55.6%)	424 (84.0%)	81 (16.0%)	315 (80.8%)	75 (19.2%)
Good	2744 (46.6%)	3146 (53.4%)	221 (77.8%)	63 (22.2%)	141 (70.5%)	59 (29.5%)

*P < 0.05

### Predictors of health services utilization

The results from multivariate logistic regression analysis in Table 4 show that predisposing factors (i.e., age, marital status), enabling factors (i.e., household income, duration of residence, range of migration, reasons for migration, size of friend network in current residence, type of health insurance, location where they obtained health insurance) and need factors (i.e., chronic diseases) were significantly associated with doctor visits. Compared to migrants aged 60–64 years, migrants aged 70 and older were more likely to see a doctor when they were ill (OR = 1.23, *P* < 0.01). Compared to the migrants with monthly household income less than 3500 yuan, migrants with household income of more than 7000 yuan were more likely to see a doctor when they were ill (OR = 1.65, *P* < 0.01). Elderly migrants who joined local health insurance were more likely to see a doctor than those who joined hometown health insurance (OR = 1.57, *P* < 0.01).

**Table 4:** Logistic regression analysis of the health services utilization among elderly migrants

Variables	Doctor visits		Hospitalization		Local inpatient care	
	OR	95% CI	OR	95% CI	OR	95% CI
Predisposing factors						
Age (year) (reference: 60–64)						
65–69	1.11*	[1.01, 1.22]	0.88	[0.57, 1.35]	1.08	[0.68, 1.71]
≥ 70	1.23**	[1.11, 1.37]	0.87	[0.57, 1.32]	1.12	[0.71, 1.76]
Female (ref: male)	1.03	[0.95, 1.11]	0.95	[0.68, 1.33]	1.23	[0.85, 1.78]
Currently married (ref: single)	0.87*	[0.78, 0.97]	1.06	[0.70, 1.61]	0.80	[0.50, 1.27]
Urban *Hukou* (ref: rural *Hukou*)	1.13	[0.99, 1.29]	0.70	[0.39, 1.27]	1.29	[0.65, 2.55]
Education (ref: elementary/less)						
Middle school	0.96	[0.87, 1.06]	0.75	[0.49, 1.14]	1.23	[0.74, 2.05]
High school/above	1.02	[0.89, 1.16]	0.81	[0.47, 1.40]	1.36	[0.75, 2.47]
*Enabling factors*						
Monthly household income (RMB) (ref:≤3500 yuan)						
3501–7000 yuan	1.29**	[1.18, 1.42]	0.91	[0.63, 1.31]	0.77	[0.49, 1.21]
> 7000 yuan	1.65**	[1.47, 1.84]	1.89*	[1.12, 3.20]	0.77	[0.46, 1.30]
Years of living in the city of residence (ref: ≤4 years)						
5–9 years	0.96	[0.88, 1.06]	1.46	[0.97, 2.20]	1.03	[0.67, 1.57]
≥ 10 years	0.88*	[0.79, 0.97]	1.36	[0.91, 2.04]	2.64**	[1.53, 4.55]
Migration range (ref: Across provinces)						
Across cities within a province	0.89*	[0.81, 0.98]	1.08	[0.73, 1.61]	1.27	[0.82,1.94]
Across counties within a city	0.85**	[0.77, 0.94]	1.31	[0.88, 1.94]	1.87**	[1.19, 2.93]
Reasons for migration(ref: on business)						
Looking after children or grandchildren	1.21**	[1.08, 1.36]	3.09**	[1.79, 5.33]	0.69	[0.34, 1.38]
Retirement/keeping fit	1.27**	[1.12, 1.43]	1.83*	[1.10, 3.05]	1.10	[0.54, 2.25]
Others	1.45**	[1.24, 1.69]	1.41	[0.73, 2.70]	0.69	[0.28, 1.68]
Friends number ≥ 5 (ref: friend number < 5)	1.23**	[1.13, 1.33]	1.03	[0.74, 1.44]	1.88**	[1.29, 2.73]
Health insurance (ref: NCMS)						
UEMI	0.79**	[0.68, 0.92]	1.86	[0.94, 3.67]	0.89	[0.42, 1.87]
Others	0.87*	[0.76, 0.99]	1.37	[0.75, 2.51]	1.63	[0.79, 3.37]
Local health insurance (ref: hometown health insurance)	1.57**	[1.38, 1.78]	1.22	[0.72, 2.05]	3.60 **	[1.47, 8.84]
Need factors						
Chronic diseases (ref: no)	1.15**	[1.05, 1.27]	1.20	[0.87, 1.67]		
Self-rated health (ref: poor)						
Neutral	1.01	[0.88, 1.15]	1.11	[0.76, 1.62]		
Good	1.14	[0.99, 1.32]	0.80	[0.51, 1.25]		
N	11,020		1128		844	
-2 Log likelihood	14,902.04		998.794		771.63	
Cox & Snell R^2^	0.025		0.037		0.095	

*P < 0.05, **P < 0.01

Household income and reasons for migration were significantly associated with hospitalization. Compared to the elderly migrants with monthly household income no more than 3500 yuan, elderly with household income of more than 7000 yuan were more likely to receive inpatient care when they needed (OR = 1.89, *P* < 0.05). Respondents who indicated descendants care (OR = 3.09, *P* < 0.01) and retirement/keeping fit (OR = 1.83, *P* < 0.05) as their migration reasons were more likely to be hospitalized than those who migrated for work.

Local inpatient care was predicted by duration of stay in the city of residence, the range of migration, friends’ number, and location where they joined health insurance. Compared to migrants with a duration of migration of less than 5 years, migration across provinces, the elderly with migration duration more than 10 years (OR = 2.64, *P* < 0.01), migration across counties within a city (OR = 1.87, *P* < 0.01) were more likely to be hospitalized in local hospitals. Elderly migrants with local health insurance were more likely to receive local inpatient care than those with hometown insurance (OR = 3.60, *P* < 0.01). Elderly respondents who had 5 or more friends in their current residence were more likely to receive local inpatient care than elderly respondents who indicated fewer than 5 local friends (OR = 1.88, *P* < 0.01).

## Discussion

In this study, we used the data from the 2015 Migrant Dynamics Monitoring Survey, and analyzed health services utilization status among elderly migrants, and we explored factors associated with the use of health services, as guided by the Behavioral Model of Health Service Use. The behavioral model focused on defining and measuring equitable health care access to develop policies and programs that promote optimal resource use. This study was of great significance in providing evidence for policy makers to develop appropriate regulations and laws to improve health care services utilization among elderly migrants.

Overall, elderly migrants used medical health services at a relatively low level compared with the general population. We found that more than half of the elderly migrants did not see a doctor when they had a minor illness, which was significantly higher than that of the general population (54.5% vs.15.5%, respectively) [21]. The annual rate of hospitalization among elderly migrants was 8.0%, smaller than half of that in the general elderly population surveyed in the Fifth National Health Service Survey (17.9%) [22]. Additionally, the proportion of migrants without hospitalization to those who were supposed to be hospitalized was 18.2%. The low level of health services utilization among elderly can be explained from two aspects. Elderly migrants could not enjoy the same social welfare and public services as local residents due to the restriction of household registration system. There was also a lack of health care awareness and health literacy among elderly migrants, leading to an underestimation of their health services needs.

The Behavioral Model of Health Service Use consists of three components, including predisposing factors, enabling factors and need factors, all of which can influence the health services utilization. Predisposing factors are not directly related to health services use, but can exert effects on health services use through enabling factors and need factors. Enabling factors are indirect influential factors while need factors are direct influential factors of health services use. In this study, we found that relative to predisposing and need factors, enabling factors contributed a lot to the health services utilization among elderly migrants.

Elderly migrants with increasing age were more likely to visit doctors with minor symptoms. It is possible that the oldest migrants were more concerned about their health. The study also indicated that a considerable number of elderly participants did not visit doctors when they suffered from diseases with minor symptoms, but they treated the illness by using the medicine bought in dispensaries or did not take any measures at all. This finding may be due to the absence of scientific health knowledge among the elderly. Usually, they did not have appropriate insight of illness symptoms, and often adopted negative attitudes toward curative services. Failure to promptly seek medical treatment caused the deterioration of illness, leading to more demands for health services [23]. The absence of health care awareness and risk perception of the elderly deserves prompt attention from health departments. More health education campaigns should be implemented to popularize health knowledge and improve health literacy.

Enabling factors consist of household income, duration of migration, migration range, reasons for migration, number of friends, and locations where they enrolled in health insurance. Elderly migrants with higher household income were more likely to visit doctors when they suffered from diseases with minor symptoms and the proportion of utilizing inpatient services was also higher compared to migrants with low income. Due to limited benefits from local social welfare, elderly migrants relied more on individual or family resources for medical services [6]. This finding was consistent with previous studies about the influence of income on health services utilization [24–26]. A study conducted among middle-aged and elderly individuals in Gansu, China, showed a pro-rich inequality in both outpatient and inpatient utilization [27]. Economic hardship has been proved to be one of the main barriers for migrants accessing health services [28]. Due to the low capability to pay for the medical services expenses, migrants usually do not choose to go to the hospital until they perceive themselves to be seriously ill, after experimenting failures with a series of ineffective health-seeking behaviors such as unsupervised self-treatment, unregulated clinics, or “just holding on” [29, 30].

Another important reason underlying the low utilization of health care services among elderly migrants was that they were not entitled to receive local health insurance and couldn’t utilize local medical resources as the urban residents do. Among migrants underinsured, 88.5% of them had health insurance from their original hometown; only 11.4% had a more accepted, local health insurance. Due to the limitations of local administration, the usage of hometown insurance in their living and working cities was faced with a series of barriers, such as inconvenient access to health services, complicated procedures, and low reimbursement rates [31]. This study also explored whether the location where elderly migrants enrolled in health insurance influenced health services utilization, and found that elderly migrants with local health insurance were more likely to visit a doctor when they were ill and choose local hospitalization. The status of health services utilization was obviously better than that of those who enrolled in their hometown health insurance. Migrants without local health insurance were limited to access medical services, which should be noted by health administration. More social welfare benefits should be provided by the government to improve elderly socioeconomic status in urban areas and lower qualifications for the elderly migrants to join the local health insurance.

The findings of the study indicate that migrants who originated from the counties within the same city, had longer duration of stay, and had more friends in the city of residence were more likely to use local inpatient services. This finding may be attributed to the fact that they had more social cohesion in their current locations and had better knowledge about local medical services [32]. Previous studies confirmed that more friends in current residence can help migrants reconstruct relatively rich social network, have greater social capital and get more functional and emotional social support [33, 34]. Elderly migrants with good social support could get access to better medical services [35]. Therefore, more community activities should be organized to strengthen the social networks of elderly migrants, assisting them in integrating into urban life and finding a sense of belonging. Few studies have been conducted to investigate the influence of migration reasons on health services utilization. In this study, we found that elderly migration for business were the more vulnerable group in accessing to health services. It was possible that the difference of health services utilization among the elderly with different reasons for migration may be related with their family economic status. The study also found that the elderly migrating to take care of descendants or live out their life in retirement usually had better family socioeconomic status.

Need factors are important factors influencing health services utilization. Two need factors, chronic disease status and self-rated health status, were investigated in the study. Elderly migrants with chronic diseases were more likely to visit doctors when they suffered from minor illnesses. People in poor health had more need for medical care and incurred more health services utilization [26, 36–38].

There are limitations in the study. First, the data used in the study were cross-sectional in nature, and no causal relationship could be detected without longitudinal data. Second, the study investigated the possibility of accessing services rather than the actual detailed behaviors of utilization. Third, the study was self-reported, and elderly migrants may have difficulties in memorizing their health services utilization for the past year. Recall bias could not be ruled out.

## Conclusions

This is the first study to apply the Behavioral Model of Health Service Use to investigate health care services use among elderly migrants in China. Elderly migrants utilized health care services at a relatively low level. Enabling factors, such as family economic status, health insurance and migration experience were important factors influencing health-seeking behaviors. Elderly migrants with low household income and without local health insurance were disadvantageous groups in utilizing local medical services. Recognizing the heterogeneity of elderly migrants, our findings recommend that policy makers may put more attention on these vulnerable populations and take targeted measures to optimize access to and utilization of health care services by elderly migrants in need of medical care.

## Abbreviations

CMIURR: Cooperative Medical Insurance for Urban and Rural Residents
NCMS: New Rural Cooperative Medical Scheme
PPS: probability proportionate to size
UEMI: Urban Employment Medical Insurance
URMI: Urban Residents Medical Insurance

## References

[1] Yu B, Chen X, Li S (2014). Globalization, cross-culture stress and health. Chinese Journal of Epidemiology.

[2] Yu B, Chen XG, Yan YQ, et al. Migration stress, poor mental health and engagement in sex with high-risk partners: A Mediation Modeling Analysis of data from rural-to-urban migrants in China. Sex Res Soc Policy. 2017;14(4):467–77. 10.1007/s13178-016-0252-y. 10.1007/s13178-016-0252-y PMC5662130 29098041

[3] The World Bank.Migration and Development Brief 28. http://www.worldbankorg/en/topic/labormarkets/brief/migration-and-remittances. Accessed 8 Nov 2017.

[4] Department of services and Management of Migrant Population National Health and Family Planning Commission of China. Report on China's migrant population development 2016. China Population Publishing House.

[5] Wu S. Forty-three percent of the floating old people migrants to take care of their children. China Population News 2016.

[6] Wang Q. Health of the elderly migration population in China: benefit from individual and local socioeconomic status? Int J Environ Res Public Health. 2017;14(4):370. 10.3390/ijerph14040370. 10.3390/ijerph14040370 PMC5409571 28368314

[7] Chen X, Yu B, Gong J (2015). The domestic migration stress questionnaire (DMSQ): development and psychometric assessment. Journal of Social Science Studies.

[8] Chen X, Yu B, Gong J, et al. Social capital associated with quality of life mediated by employment conditions: Evidence from a random sample of rural-to-urban migrants in China. Soc Indic Res. 2017:1–20. 10.1007/s11205-017-1617-1. 10.1007/s11205-017-1617-1 PMC6110534 30166770

[9] Chen XG, Stanton B, Kaljee LM (2011). Social stigma, social capital reconstruction, and rural migrants in urban China: a population health perspective. Hum Organ.

[10] Quande Z. The supporting problem of elderly migrants is worth attention. China Population News 2016–10-17;003.

[11] Kochar A (1999). Evaluating familial support for the elderly: the intrahousehold allocation of medical expenditures in rural Pakistan. Economic Development & Cultural Change.

[12] Rechel B, Grundy E, Robine JM, et al. Ageing in the European Union. Lancet. 2013;381(9874):1312–22. 10.1016/s0140-6736(12)62087-x. 10.1016/S0140-6736(12)62087-X 23541057

[13] Hesketh T, Jun YX, Lu L (2008). Health status and access to health care of migrant workers in China. Public Health Rep.

[14] Li Y. Understanding health constraints among rural-to-urban migrants in China. Qual Health Res. 2013;23(11):1459–69. 10.1177/1049732313507500. 10.1177/1049732313507500 24122513

[15] Gong P, Liang S, Carlton EJ (2012). Urbanisation and health in China. Lancet.

[16] Mou J, Cheng JQ, Zhang D, et al. Health care utilisation amongst Shenzhen migrant workers: does being insured make a difference? BMC Health Serv Res. 2009;9:9. 10.1186/1472-6963-9-214. 10.1186/1472-6963-9-214 PMC2788549 19930580

[17] Andersen R, Aday LA (1978). Access to medical care in the U.S.: realized and potential. Med Care.

[18] Guo J, Zhou QY, Weng HY (2015). Analysis on multilevel logistic regression model of the utilization of health services for migrant population and influencing factors. Chinese Health Economics.

[19] Park JM. Equity in the utilization of physician and inpatient hospital services: evidence from Korean health panel survey. Int J Equity Health. 2016;15(1):159. 10.1186/s12939-016-0452-3. 10.1186/s12939-016-0452-3 PMC5041589 27681229

[20] Alemi Q, Stempel C, Koga PM, et al. Determinants of health care services utilization among first generation afghan migrants in Istanbul. Int J Environ Res Public Health. 2017;14(2). 10.3390/ijerph14020201. 10.3390/ijerph14020201 PMC5334755 28218688

[21] Xu L, Qun M (2014). The results of the Fifth National Health Service Survey——health service needs, demands and utilization. Chinese Journal of Health Informatics and Management.

[22] National Health and Family Planning Commission of the People's Republic of China. An Analysis Report of National Health Services Survey in China, 2013.

[23] Peng YC, Chang WH, Zhou HQ, et al. Factors associated with health-seeking behavior among migrant workers in Beijing, China. BMC Health Serv Res. 2010;10(1):1–10. 10.1186/1472-6963-10-69. 10.1186/1472-6963-10-69 PMC2848137 20298613

[24] Xie E (2011). Income-related inequalities of health and health care utilization. Frontiers of Economics in China.

[25] Li CF, Dou L, Wang HP, et al. Horizontal inequity in health care utilization among the middle-aged and elderly in China. Int J Environ Res Public Health. 2017;14(8):842. 10.3390/ijerph14080842. 10.3390/ijerph14080842 PMC5580546 28933772

[26] Amente T, Kebede B. Determinants of health service utilization among older adults in Bedele Town, Illubabor Zone, Ethiopia. J Diabetes Metab Disord. 2016;7(11):7. 10.4172/2155-6156.1000713.

[27] Wang Y, Wang J, Maitland E (2012). Growing old before growing rich: inequality in health service utilization among the mid-aged and elderly in Gansu and Zhejiang provinces, China. BMC Health Serv Res.

[28] Zhanxin Zhang, Hou Y. Floating population in urban communities: Social Sciences Academic Press, 2009.

[29] Zou G, Zeng Z, Chen W, et al. Self-reported illnesses and service utilisation among migrants working in small-to medium sized enterprises in Guangdong, China. Public Health. 2015;129(7):970–8. 10.1016/j.puhe.2015.04.015. 10.1016/j.puhe.2015.04.015 26077388

[30] Hong Y, Li X, Stanton B (2006). Too costly to be ill: health care access and health seeking behaviors among rural-to-urban migrants in China. World Health Popul.

[31] Luo X. Realizing remote medical insurance is the only way leading to health care reform. Health Economics Research. 2011;(09):17–9. 10.14055/j.cnki.33-1056/f.2011.09.006

[32] Liu S, CXJ H, Mak S. Comparison of health status and health care services utilization between migrants and natives of the same ethnic origin-the case of Hong Kong. Int J Environ Res Public Health. 2013;10(2):606–22. 10.3390/ijerph10020606. 10.3390/ijerph10020606 PMC3635166 23435588

[33] Chen X, Stanton B, Gong J, et al. Personal social capital scale: an instrument for health and behavioral research. Health Educ Res. 2009;24(2):306–17. 10.1093/her/cyn020. 10.1093/her/cyn020 18469318

[34] Wang PG, Chen XG, Gong J, et al. Reliability and validity of the personal social capital scale 16 and personal social capital scale 8: two short instruments for survey studies. Soc Indic Res. 2014;119(2):1133–48. 10.1007/s11205-013-0540-3.

[35] Wang JF, Wu YY, Zhou BA, et al. Factors associated with non-use of inpatient hospital care service by elderly people in China. Health Soc Care Community. 2009;17(5):476–84. 10.1111/j.1365-2524.2009.00849.x. 10.1111/j.1365-2524.2009.00849.x 19456901

[36] Li Y, Chi I, Zhang K (2006). Comparison of health services use by Chinese urban and rural older adults in Yunnan province. Geriatr Gerontol Int.

[37] Kouzis AC, Eaton WW (1998). Absence of social networks, social support and health services utilization. Psychol Med.

[38] Song XL, Zou GY, Chen W, et al. Health service utilisation of rural-to-urban migrants in Guangzhou, China: does employment status matter? Tropical Med Int Health. 2017;22(1):82–91. 10.1111/tmi.12801. 10.1111/tmi.12801 27775826

